# Both the Complexity of Tight Junctions and Endothelial Transcytosis Are Increased During BBB Postnatal Development in Rats

**DOI:** 10.3389/fnins.2022.850857

**Published:** 2022-04-28

**Authors:** Wei Li, Jinlong Zou, Junkui Shang, Chenhao Gao, Ruihua Sun, Ruijie Liu, Huixia Cao, Yanliang Wang, Jiewen Zhang

**Affiliations:** ^1^Department of Neurology, Zhengzhou University People’s Hospital, Henan Provincial People’s Hospital, Zhengzhou, China; ^2^Department of Neurology, Henan University People’s Hospital, Henan Provincial People’s Hospital, Zhengzhou, China; ^3^Department of Nephrology, Henan Provincial Key Laboratory of Kidney Disease and Immunology, Henan Provincial People’s Hospital, Zhengzhou University People’s Hospital, Henan University People’s Hospital, Zhengzhou, China

**Keywords:** blood-brain barrier, transcytosis, tight junctions, RNA-seq, caveolin-1

## Abstract

The blood-brain barrier (BBB) comprises a single layer of endothelial cells and maintains a safe and homeostatic environment for proper neuronal function and synaptic transmission. BBB is not a discrete physical barrier, but a complex, dynamic, and adaptable interface. BBB continues to mature under the influence of the neural environment within a short period of time after birth. However, the basic mechanism of BBB formation and maintenance remains a mystery. Early studies have identified two structural characteristics of microvascular endothelium: special tight junctions (TJs) and a very low transcellular vesicle transport rate. Previous studies believed that BBB damage was mainly due to the destruction of tight junctions, and the role of vesicle transcytosis was neglected, so there was a lack of research on its impact on blood-brain barrier. It is urgent to get a better clarification of the unique structural and functional characteristics of the BBB endothelium to explain the role of BBB injury in neurological diseases. RNA sequencing was used to study the molecular characterization of cerebral cortex vascular endothelium by isolating them from neonatal, adolescent and adult rats. For investigation the maintenance mechanism of the BBB, we focused on the cellular and molecular regulation of barrier formation and the two characteristics of microvascular endothelial cells. Interestingly, we found that during the development of the blood-brain barrier, although the tight junctions gradually mature, endothelial cell transcytosis is gradually enhanced, resulting in an increase in the permeability of the blood-brain barrier. This study suggested that under physiological conditions, low vesicle transport is playing an important role in maintaining the integrity of the blood-brain barrier. This study not only summarized the unique characteristics of microvascular endothelial cells, but also illustrated a clarified mechanism of the development and maintenance of BBB which can provide new therapeutic opportunities for central nervous system drug delivery. Raw data of RNA sequencing were deposited in NCBI Sequence Read Archive database (PRJNA790676).

## Graphical Abstract

Graphical representation of the proposed molecular pathway. During BBB development, cav-1 is gradually upregulated, vesicle transcytosis is gradually increased. Although tight junctions are gradually matured, BBB permeability is still increased, revealing the important role of low vesicle transcytosis in maintaining BBB permeability.

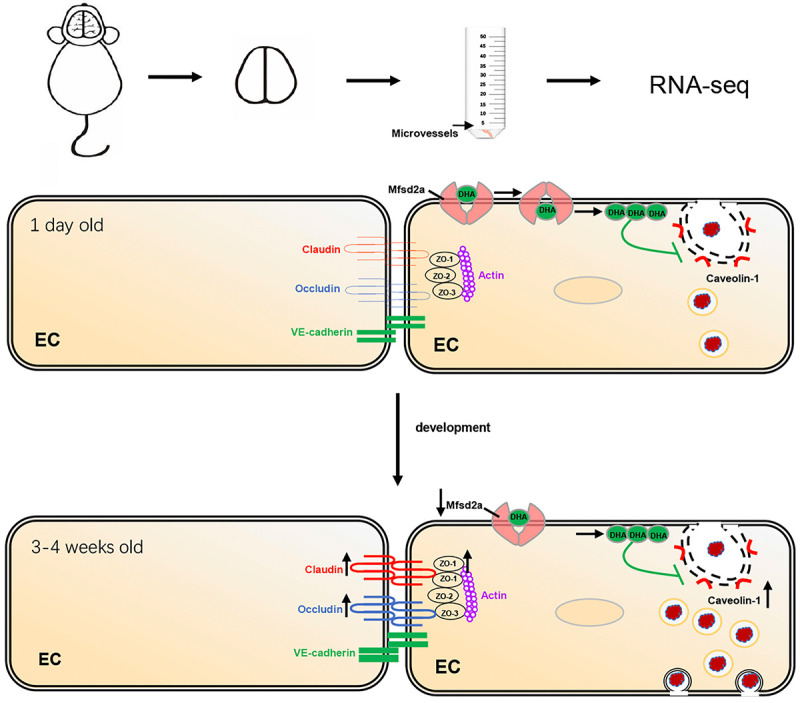

## Introduction

The blood-brain barrier (BBB) is the interface that separates neural tissue from circulating blood, which comprises a single layer of endothelial cells that forms the blood vessel wall and maintains a safe and homeostatic milieu for proper neuronal function and synaptic transmission (Van Dyken and Lacoste, 2018; Langen et al., 2019). Impaired barrier function is associated with neurodegenerative diseases, including multiple sclerosis, Alzheimer’s disease and Parkinson’s disease (Zhen et al., 2015; Sweeney et al., 2018). The BBB also acts as an obstacle for the treatment of neurological disorders as it can greatly limit drug delivery into the brain (Pardridge, 2007). In addition, the BBB is not a discrete physical barrier but a complex, dynamic and adaptable interface (Ballabh et al., 2004; Hirohata et al., 2015; Yu et al., 2020). Barrier genesis in mice occurs between days E10 and E15, and the BBB continues to mature under the influence of neural environment over a brief period after birth, however, the fundamental mechanisms underlying the formation and maintenance of the BBB remain a mystery (Hagan and Ben-Zvi, 2015; Zhen et al., 2015).

Early research identified two structural features of microvascular endothelium that underlie the restrictive properties of the BBB (Haseloff et al., 2015). First, the endothelial cells of the central nervous system (CNS) have special tight junctions (TJs) to prevent free cell-side channels from passing through the blood vessel wall (Farquhar and Palade, 1963; Reese and Karnovsky, 1967). Second, they exhibit a very low rate of transcellular vesicle transport, or transcellular transcytosis, to limit transcellular transport through the blood vessel wall (Andreone et al., 2017). It was previously assumed that the maintenance of the BBB was mainly due to TJs (Siegenthaler et al., 2013). The role of vesicular transcytosis has been overlooked, therefore, there is a lack of research on its influence on BBB integrity. The mechanisms regulating this inhibition at the BBB were largely unknown until recent studies established the domain-containing protein 2a (mfsd2a) as an inhibitor of transcytosis, which is critical for BBB formation and maintenance (Andreone et al., 2017). Mfsd2a establishes a unique lipid environment, which inhibits caveolae vesicle formation in CNS endothelial cells to suppress transcytosis and ensure BBB integrity (Ben-Zvi et al., 2014). In the brain, caveolin-1 (cav-1) is the major component of caveolae, and it is primarily expressed in endothelial cells (Anderson, 1998). With a deeper knowledge of the unique structural and functional characteristics of the BBB endothelium, we would get a significantly better understanding of the contribution of the BBB impairment to neurological disorders.

The phenotypic characteristics of the brain barrier are ultimately regulated at the transcriptional level. There were several studies that used isolated brain endothelial cells of mice and performed various genomic sequencing. Their results provide a useful resource for the BBB research field and shed some insights into the development and biology of BBB. By evaluating the gene expression profile of endothelial cells from mouse brain cortices, Tam et al. (2012) identified DR6/TNFRSF21 and TROY/TNFRSF19 as regulators of CNS specific angiogenesis in both zebrafish and mice. Sabbagh et al. (2018) explored transcriptome differences between CNS and non-CNS vascular endothelial cells from P7 mouse neonates. They performed RNA-seq to determine the factors that are associated with endothelial cell heterogeneity (Sabbagh et al., 2018). These studies provide an integrated view of the genetic and epigenetic mechanisms underlying the unique tissue-specific programs of endothelial cell differentiation. A recent study used fluorescence-activated cell sorting (FACS) on brain, liver, lung, kidney, and cardiac endothelial cells from adult mice and performed RNA-seq, suggesting a common mechanism for BBB dysfunction throughout different neurological disorders (Munji et al., 2019). These resources may provide important information about the mechanisms by which CNS endothelial cells develop their unique properties, how these properties are disrupted during injury and disease, and to study novel targets that modulate the BBB and deliver drugs through the BBB to treat different neurological diseases.

Several studies have applied different genomics to provide information about CNS vascular gene expression, but they have shown three limitations. First, since these studies used whole capillaries that contain many different cell types, the purity of microvascular endothelial cells is unknown (Enerson and Drewes, 2006). Second, most of the published studies lack an in-depth description of the CNS endothelial isolation procedures (Richard et al., 2010). Third, these studies used the transcriptome of endothelial cells from the entire brain instead of microvascular endothelial cells (Munji et al., 2019). Working to address these shortcomings, we attempted to characterize the molecular signature of the cerebrovascular endothelium by transcriptome and the profiling of isolated cerebral cortical endothelial cells, focusing on the cellular and molecular regulation of barrier formation and the maintenance of BBB. The purpose of this study was to clarify the temporal profile of BBB TJs, the development of endothelial cell transcytosis and the signaling pathways driving this process. This study not only summarized the unique characteristics of microvascular endothelial cells, but also illustrated a clarified mechanism of the development and maintenance of BBB which can provide new therapeutic opportunities for CNS drug delivery.

## Materials and Methods

### Study Design

One-day-old, 3–4-week-old (80 ± 10 g) and 6-month-old (360 ± 10 g) male SD rats were used in this study. Three biological replicates at each time point were used for sequencing. In one-day-old group, there are 10 rats per repetition, in 3–4-week-old and 6-month-old groups, there are 5 rats per repetition. Previous studies suggested sex-specific differences in tight junction protein expression that could affect BBB function (Weber and Clyne, 2021). To avoid the effect of gender on the blood-brain barrier, we mainly studied the maintenance and development of the blood-brain barrier in male rats. All animal experiments were approved by the Ethics Committee of Henan Provincial People’s Hospital, Zhengzhou University, China, and every effort was made to minimize the number of animals and their suffering. All animals were in a light-and temperature-controlled environment with access to food and water *ad libitum*. In order to minimize the subjective bias in the design, we adopt the principles of randomization and double blind.

### Measurement of Blood-Brain Barrier Permeability

Evans Blue (EB) extravasation was used to assess BBB permeability (Lobo et al., 2020). In brief, 2% EB (3 mL/kg, Sigma) was injected *via* the vein at various timepoints. After the EB was allowed to circulate for 2 h, the rats were anesthetized and then perfused transcardially with normal saline solution. To assess EB extravasation, brain cortices were isolated and weighed, homogenized in 1 ml of 50% trichloroacetic acid, and centrifuged at 10,000 rpm for 30 min. The supernatant was collected and mixed with an equal amount of ethanol. EB concentrations were determined spectrophotometrically at 620 nm. The permeability of the blood-brain barrier was evaluated by calculating the EB content according to the standard curve.

### Endothelial Cell Enrichment

After a rat was anesthetized with isoflurane, the head was washed with 70% alcohol, and quickly separated from the body to remove the brain. Then, the thin slices of subdural meninges (SDM) on the dorsal surface of the brain were carefully removed using the dissecting microscope to validate the removal. The brain was cut in half horizontally and divided cautiously into cortex and internal structures, and dissected in cold phosphate-buffered saline (PBS). The separated cerebral cortex was properly cut into 1 mm^3^ pieces of tissue in order to facilitate enzyme-based digestion or the subsequent Dounce homogenization steps. The tissue pieces were incubated in type II collagenase (Gibco 17101015, 1 mg/mL) and 100 μL DNase1 to DMEM F12. Microvascular pellets were resuspended in a 20% BSA-V solution and centrifuged (4°C) for 8 min at 1,000 × *g* to remove the top layer, containing myelin and brain parenchymal cells. Then, the microvascular pellets were filtered with a 40 μm cell strainer and centrifuged at 1,000 × *g* for 2 min to obtain pure pink microvascular fragments. The microvascular fragments were directly sorted into 1 mL trizol (Invitrogen 15596018), and stored at −80°C with liquid nitrogen.

### RNA Extraction, Library Construction and Sequencing

After the total RNA was extracted using a trizol reagent kit (Invitrogen, Carlsbad, CA, United States) according to the manufacturer’s instructions, all rRNAs were removed to retain the mRNAs. The remaining clean reads were further used in assembly and gene abundance calculation. The quality and integrity of total RNA were assessed using Nano Photometer spectrophotometer (IMPLEN) and monitored on 1% agarose gel. The purified mRNA was cut into short fragments using fragmentation buffer and reverse transcribed into cDNA with random primers. RNA concentration was measured with the Qubit RNA Assay Kit in a Qubit 2.0 fluorometer (Life Technologies). The ligation product was amplified and enriched PCR to construct a cDNA library template. The ligation products were size selected by agarose gel electrophoresis, PCR amplified, and sequenced using Illumina Novaseq6000 by Gene *de novo* Biotechnology Co., Ltd., (Guangzhou, China). Raw data of RNA sequencing were deposited in NCBI Sequence Read Archive database (PRJNA790676).

### Quantitative Reverse Transcription Polymerase Chain Reaction

After the total RNA was extracted from different development of brain cortex microvessels using a trizol reagent kit (Invitrogen, Carlsbad, CA, United States), expression levels of ocln, cldn5, tjp1 genes were compared using real-time quantitative PCR (qRT-PCR). Perform three to six repetitions for each sample (5–6 rats/repetition). cDNA (500 ng) was synthesized from obtained RNA samples using a reverse transcription kit (HiScript II Q RT SuperMix for qPCR, Vazyme). Quantitative PCR reactions were prepared using ChamQ Universal SYBR qPCR Master mix (Vazyme) and specific primers (Table 1) on the ABI 7500 system (Applied Biosystem). qRT-PCR thermal cycle reaction started at 95°C for 5 min, followed by 40 cycles of 95°C for 10 s, 60°C for 30 s. Relative gene expression in ocln, cldn5, tjp1 was calculated using the 2−ΔΔCt method (Livak and Schmittgen, 2001).

**TABLE 1:** Oligonucleotide primer sequences for qRT-PCR.

Gene	Primer direction	Sequence (5′–3′)
GAPDH	Forward	TGCCACTCAGAAGACTGTGG
	Reverse	TTCAGCTCTGGGATGACCTT
Cldn5	Forward	TCACAGAGAGGGGTCGTTGA
	Reverse	CAGGTTAGCAGGTACCACCG
Ocln	Forward	ATTGAGCCCGAGTGGAAAGG
	Reverse	CACAGAGGTAGCACCACGTT
Tjp1	Forward	GGACCCTCCTCAGACCTTCT
	Reverse	TCACAGTGTGGCAAGCGTAG

### Western Blotting

Rats were sacrificed under anesthesia through transcardiac perfusion with cold phosphate-buffered saline (PBS). We extracted rat cerebral cortical microvessels (groups of 5–6 rats with three replicates each), weighed and homogenized the microvessels in RIPA lysis buffer. Samples were homogenized, and protein concentrations were determined by bicinchoninic acid (BCA) protein assay (Thermo-Fisher, Waltham, MA, United States). The proteins were separated on 10% SDS–PAGE gel and transferred to nitrocellulose membranes (Invitrogen, United States). After three times of washing in TBS with 0.1% Tween-20 (TBST), the membranes were blocked in TBST with 5% skimmed milk for 2 h at room temperature. The membranes were incubated overnight with primary antibodies at 4°C and then further incubated with HRP-conjugated secondary antibodies (1:1,500) for 1 h at room temperature. The following primary antibodies were used: rabbit anti-occludin (1:100, Thermo Fisher Scientific, #711500); rabbit anti-claudin 5 (1:100, Thermo Fisher Scientific, # 34–1600); rabbit anti-zo-1 (1:100, Thermo Fisher Scientific, #402200) and rabbit anti-caveolin-1 (1:1,000, Cell Signaling Technology, #3238). The secondary antibodies were used: goat anti-rabbit IgG (1:1,500, Beyotime Biotechnology, #A0208). Western Bright ECL solution was used to develop the blots. Chemiluminescence antibody signals were documented using Chemidocit 510 image system (Analytik Jena United States) and Vision Works software (Media Cybernetics, Rockville, MD, United States), using exposure settings and quantified for band densitometry. Each membrane was then probed for β-actin (1:2,000, Beyotime Biotechnology, #AF5003) as endogenous loading controls. The antibody used to probe for occludin, claudin5, zo-1 and caveolin-1 detect the same bands as reported in previous publications. Relative protein expression was quantified by measuring band densitometry. Images for the blots of a protein of interest and their corresponding β-actin were selected for analyses and presentation based on a predetermined upper grayscale value to assure linear densitometric values. Images were analyzed using the lane and band functions of the ImageLab™ software. For presentation purposes, the grayscale range was set between 0 units and the predetermined upper grayscale value for the protein of interest and corresponding β-actin prior to exporting as an image file. Post-export modifications of the images were limited to cropping to the regions of interest.

### Transmission Electron Microscope

After the right hemisphere was divided into 1-mm^3^ sections, some tissue close to the bregma was selected and further cut into 1 × 1 × 1 mm tissue blocks. The tissue blocks were incubated with 2.5% glutaraldehyde for 6 h, dehydrated, and embedded in epoxy resin. Ultrathin sections were cut at 60 nm thickness and observed under an electron microscope (Hitachi TEM system, Tokyo, Japan).

### Statistical Analysis

Comparisons between multiple groups were made by one-way ANOVA followed by Dunnett’s *post hoc* test. Data normality was assessed using the Shapiro–Wilk test. The data were presented as mean ± SD, and the boxplots show the maximum and minimum values. Data were considered significant when *p < 0.05, ^**^p < 0.01, ^***^p < 0.001 and ^****^p < 0.0001.

## Results

### Cortical Microvascular Enrichment for RNA-Sequencing

**FIGURE 1:**
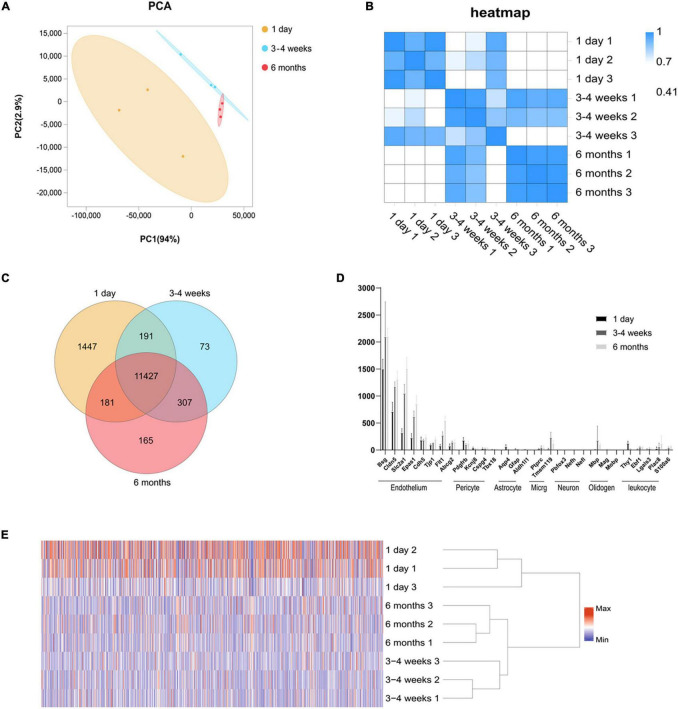
Global gene expression profiling among three cerebral microvascular groups. **(A)** Principal component analysis (PCA) of RNA-seq data from three groups of brain microvessels. The percentage refers to the contribution of this principal component to the overall variance. The distance between the points reflects the difference between the samples they correspond to. **(B)** Transcript-based sample Pearson’s correlation analysis shows the transcript expression level correlation among neonatal (1-day-old), adolescent (3–4-week-old) and adult (6-month-old) groups. The abscissa indicates the sample name, the ordinate indicates the corresponding sample name, and the color indicates the correlation coefficient size. **(C)** Venn diagram of differentially expressed transcripts among three groups. **(D)** Comparison of the expression levels (FPKM) of endothelial cell markers and non-endothelial cell markers in endothelial cells enriched from microvessels of the brain. The brain vascular samples in 1-day-old (black bars), 3–4-week-old (gray bars) and 6-month-old (French gray bars) rats are enriched with RNA of markers for CNS endothelial cells but not enriched with RNA of markers for CNS pericytes, astrocytes, microglia (micrg), neurons, oligodendrocytes (oligoden) and leukocyte. **(E)** The heat map of cluster analysis shows the whole transcript expression levels among three cerebral microvascular groups. The color scale illustrates the relative expression levels across all samples: red represents an expression level above the mean, and blue represents an expression level lower than the mean. The dendrogram on the left of the heat map shows the clustering of the transcripts. Data are presented as mean ± SEM; *n* = 5–10 per group.

Cortical microvessels have an important regulatory function for the maintenance of the brain microenvironment. To further understand the functional status of the cortical microvessels in different development periods, we enriched the cortical microvessels and then performed RNA sequencing. To isolate microvessels from the rat cerebral cortex, as shown in Supplementary Figure 1, we adapted and modified the protocol initially described by Zlokovic (Wu et al., 2003; Lee et al., 2019; Ujifuku et al., 2020). In brief, using type II collagen digestion, resuspension and 40 μm cell filter filtration, we enriched cortical microvessels. After the enrichment of microvascular endothelial cells from the cerebral cortex, we isolated the RNA for sequencing. The cortical microvascular samples showed high levels of RNA from endothelial cell genes with minimal levels of RNA from neuronal and glial genes. The level of mural cell genes in the cerebral microvascular samples was low but detectable, and estimated to be < 2.0% of the RNA in the 3–4 weeks cerebral microvascular samples (Figure 1D).

### Overview of the Landscape of Rat Cortical Microvascular Transcriptome From Neonatal to Adult

We performed RNA-seq following the cortical microvascular purification at three different developmental stages for the neonatal (1-day-old), adolescent (3–4-week-old) and adult (6-month-old) groups with three biological replicates per group (5–10 rats per biological replicate). The RNA-seq generated an average of 5.9 billion reads per sample, and more than 93% of the reads were mapped to the rat genome. The cortical microvascular samples showed high levels of RNA from endothelial cell genes with minimal levels of RNA from neuronal and glial genes. The mural cell genes had low but detectable level, which was estimated to be < 2.0% of the RNA in the 3–4 weeks cerebral microvascular samples (Figure 1D). We used principal component analysis (PCA), hierarchical clustering and heat map based on Pearson’s correlation analysis between different samples to make transcriptome-wide, unbiased comparisons between neonatal, adolescent and adult groups. In PCA, adolescent and adult datasets also clustered together along the principal component (PC1, explaining 94% of the variance; and PC2, explaining 2.9% of the variance), while weaker clustering along the principal component (PC1, explaining 94% of the variance; and PC2, explaining 2.9% of the variance) was observed for the neonatal datasets (Figure 1A). Altogether, in hierarchical clustering, these results suggested that neonatal datasets have more variability than adolescent and adult samples, potentially attributable to differences in genetic background, or brain regions which are harder to separate (Figure 1E). In the heat map, we observed a high level of measurement consistency among biological replicates, although neonatal datasets had more variability (Figure 1B). The heatmap demonstrated that “3–4 weeks-3” was intermediate between 1-day-old groups and other 3–4-week groups. 3–4 weeks old rats were used at this time point, “3–4 weeks-3” rats were 3 weeks old rats, “3–4 week-2” and “3–4 week-1” were 4 weeks old rat, which may lead to differences in intra-group. The transcriptome data revealed that the neonatal group was significantly different from the adult group, however, there were small differences in the transcriptome data between the adolescent group and the adult group (Figure 1B). To quickly find common and unique genes among different groups, we performed Venn diagram analysis. There were 11,427 overlapping genes among three groups in the Venn diagram, and the most unique changes were observed in the neonatal group (Figure 1C).

### Cortical Microvascular Transcriptional State at Three Developmental Stages

**FIGURE 2:**
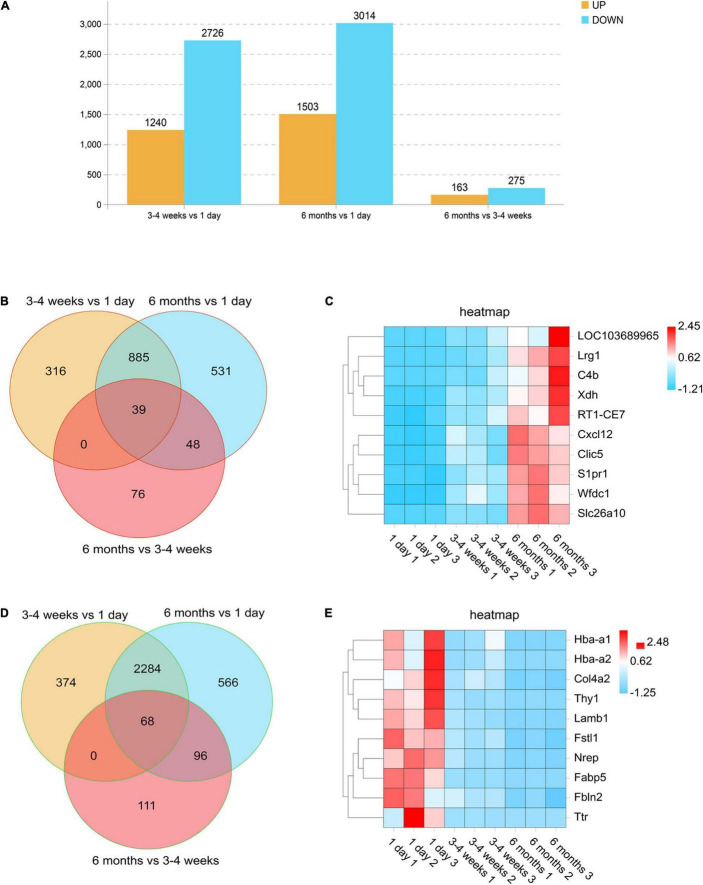
Differentially expressed genes in rat microvessels among the three groups. **(A)** Histogram analysis comparing the number of upregulated and downregulated genes between each group. Orange represents genes that are up-regulated during growth and development, and blue represents different genes that are down-regulated during growth and development. **(B,D)** Venn diagrams of the numbers of upregulated **(B)** and downregulated **(D)** gene changes in the microvascular endothelial cells observed at all-time points. For each time point, genes were selected as upregulated if they were changed by log2 (fold change) > 2.00, with FDR < 0.05 and downregulated if they were changed log2 (fold change) > –2.00, with FDR < 0.05. **(C,E)** Heat maps illustrating the transcript abundance in biological triplicates among three microvascular groups for the 10 highest confidence 6-month-old enriched **(C)** and 1-day-old enriched **(E)**. The color scale shows the relative expression levels across all samples: red represents an expression level above the mean, and blue represents an expression level lower than the mean.

In order to compare the changes in gene expression among each development stage, we defined the numbers of upregulated genes (log2 ratio > 2.000; *F* < 0.05) and downregulated genes (log2 ratio < −2.000; *F* < 0.05) at each stage. We used a histogram to analyze the number of upregulated and downregulated genes in each comparison group. As seen in Figure 2A, the cortical BBB related genes of rats in the neonatal group and the adolescent group are quite different. Moreover, compared with the neonatal group, the number of downregulated genes in rats of the adolescent group was higher than the number of upregulated genes. However, the BBB-related genes had little change between the adolescent group and the adult group, which indicates that there is no significant difference in the BBB between adolescence and adulthood. Surprisingly, the number of upregulated genes was more than that of the downregulated genes during BBB maturation. Venn diagram analysis also was conducted to find the gene expression changes during development. We observed that the expression of 39 genes remained elevated with age, such as Cxcl12, which plays an important role in development (Figures 2B,C). Furthermore, there were 68 genes whose expression levels continued to decrease, such as Ttr (transthyretin) (Figures 2D,E). As a new transporter of nanoparticles for receptor-mediated transcytosis in rat brain microvessels, transthyretin may contribute to neonatal administration to penetrate the BBB (Kim et al., 2015). These differences indicate several well-known neonatal-specific mechanisms that are different from those in adults, which reflects the adaptation to a neonatal-specific environment. The heat maps illustrate the transcript abundance in biological triplicates among three microvascular groups for the 10 highest confidence in 6 months-enriched (Figure 2C) and 1 day-enriched (Figure 2E).

**FIGURE 3:**
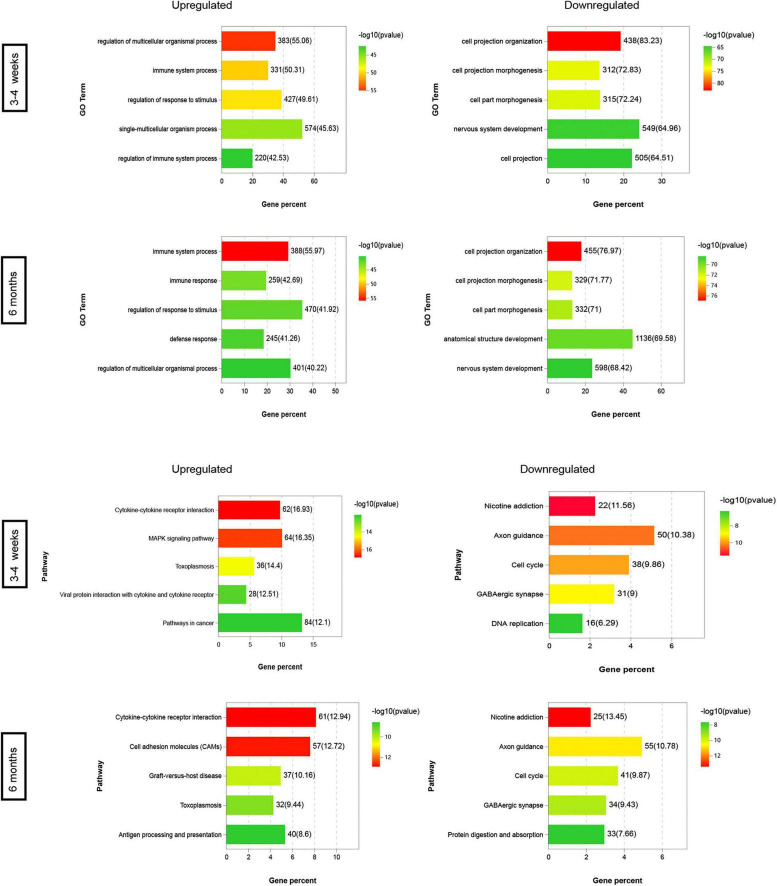
Pathways altered in the microvascular endothelial cells during development. DAVID Bioinformatics were used to identify the most significantly upregulated and downregulated GO terms and KEGG pathways based on the transcriptional changes in microvascular endothelial cells at each time point during development. For the 3–4-week-old group and the 6-month-old group, genes were selected as upregulated if they are increased compared to 1-day-old group log2 (fold change) > 2.00, with FDR < 0.05, and downregulated compared to 1-day-old group if they are changed log2 (fold change) > –2.00, with FDR < 0.05. The heat map scale represents the top5 of the *P* value that the specified pathway is changed in the given time point during development. The top five of each of the GO terms and KEGG pathways are presented at each time point during development.

We identified the signaling and biological pathways altered during brain development at each stage by DAVID Bioinformatics (Figure 3). The pathways which were altered in each stage, as well as unique pathways, were shared. Examples of upregulated pathways in the 3–4-week-old group were “regulation of multicellular organismal process,” “immune system process,” “regulation of response to stimulus,” “single-multicellular organism process,” and “regulation of immune system process,” as identified by Gene Ontology (GO). This showed that the ability of the BBB to adapt to the environment gradually increases with age. Strikingly, in the 6-month-old group, the top 3 upregulated GO pathways were “immune system process,” “immune response,” and “regulation of response to stimulus.” Downregulated pathways were the most unique to “cell projection organization,” “cell projection morphogenes,” “cell part morphogenes,” and “nervous system development” both in the 3–4-week-old and 6-month-old groups. The most unique upregulated Kyoto Encyclopedia of Genes and Genomes (KEGG) pathways in the 3–4-week-old and 6-month-old groups were “cytokine-cytokine receptor interaction.” We observed that the top 3 downregulated KEGG pathways were “nicotine addiction,” “axon guidance” and “cell cycle” both in the 3–4-week-old and the 6-month-old groups.

### Change in Microvascular Endothelial Transcytosis During Blood-Brain Barrier Development

**FIGURE 4:**
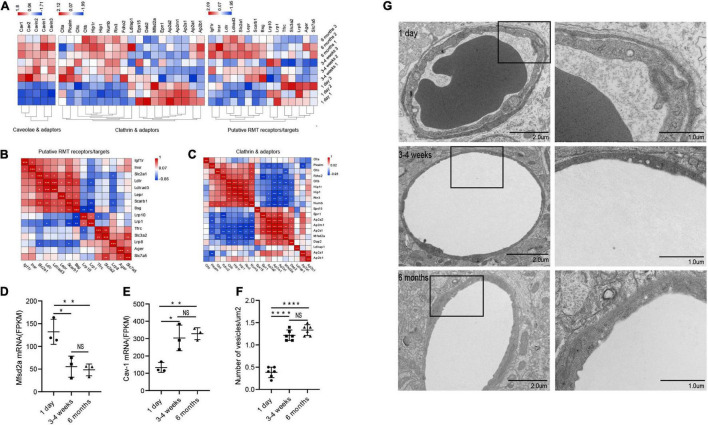
Changes in microvascular endothelial transcytosis during development. **(A)** Microvascular endothelium gene expression of putative RMT receptors, clathrin adaptors and caveolae and caveolae inhibitor (CI). **(B,C)** The correlation heat map analysis of putative RMT receptors and clathrin adaptors. **(D)** The bar graph shows the expression trend of mfsd2a in RNA transcriptome sequencing (*n* = 3). **(E)** The bar graph shows the expression trend of cav-1 in RNA transcriptome sequencing (*n* = 3). **(F)** Quantification of the number of vesicles in EC (*n* = 6). **(G)** Representative images of the ultrastructure of the microvasculature in the 1-day-old, 3–4-week-old and 6-month-old groups. Mean values ± SD, NS, not significant; *p < 0.05, **p < 0.01, ***p < 0.001 and ****p < 0.0001 compared to the Sham group; one-way ANOVA followed by Dunnett’s *post hoc* test.

**FIGURE 5:**
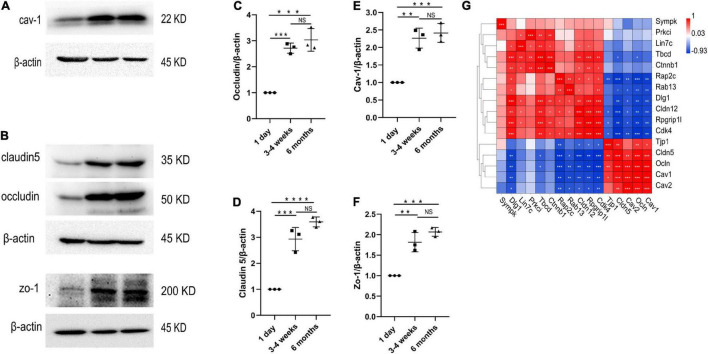
Changes in protein levels of TJs and cav-1 in the cerebral cortex microvessels. **(A–F)** Western blotting analysis and quantification of cav-1, zo-1, claudin5 and occludin expression in the cortex microvessles at three different time points (*n* = 3); Mean values ± SD, NS, not significant; *p < 0.05, **p < 0.01, ***p < 0.001 and ****p < 0.0001 compared to the Sham group; one-way ANOVA followed by Dunnett’s *post hoc* test. **(G)** Correlation heat map analysis of cav-1, cav-2 and TJs genes.

Principally, maintaining the integrity of the BBB is one of the essential functions of microvessels in the brain. We further analyzed the gene modules related to BBB function. During development, BBB demonstrates some important differences, particularly in transport mechanisms, many of which have only recently been described. Transcytosis in microvascular endothelial cells can be divided into two categories: receptor-mediated transcytosis (RMT), in which the ligand binds to the receptor to mediate endocytosis, and non-selective adsorbent transcytosis (Villaseñor et al., 2017). The two main endocytic pathways of the BBB are the clathrin-mediated pathway and the caveolin-mediated pathway (Villaseñor et al., 2017). We further observed the changes of RMT receptors, clathrin adaptors and caveolin-adaptors during the development of BBB. First, over time, the expression of some RMT receptors and clathrin junctions increases with age, however, that of the remaining genes decreases with age (Figure 4A). To investigate the correlation of RMT receptor genes, we performed a correlation heat map analysis. We observed scarb1 expression parallel to other correlates, such as slc2a1, bsg, ldlr, igf1r, lepr, insr, forming a co-expression module that suggests common orchestration. The expression of this scarb1 module was mutually exclusive with respect to that of tfrc, lrp8, slc3a2, slc7a5, and Ager (Figure 4B). Interestingly, in the correlation analysis of clathrin-related genes, we found the same phenomenon. We observed Cltb expression compared with other correlates, such as Rin3, Hip1r, Hip1, forming a co-expression module. The expression of this Cltb module was mutually exclusive with respect to the expressions of epn1, ap2a2, ap2s1 (Figure 4C). This showed that there were different dominant RMT receptors and clathrin adaptors between the neonatal and the adult group, which may provide novel therapeutic opportunities for CNS drug delivery. Next, we investigated the expression of cav1, which encodes the principal component of caveolae. The result showed that it trended upward with age (1 day vs 3–4 weeks *P = 0.0138, df = 6; 1 day vs 6 months ^**^p = 0.0074, df = 6), to a mechanism of BBB development (Figure 4E). Meanwhile, mfsd2a expression decreased (1 day vs 3–4 weeks *P = 0.0126, df = 6; 1 day vs 6 months ^**^p = 0.0083, df = 6), the product of which suppresses caveolae formation (Figure 4D). We next separated the cerebral cortex microvessels of rats at three time points and extracted the proteins for Western blot analysis. We found that the expression of cav-1 protein gradually increased (1 day vs 3–4 weeks ^**^P = 0.0012, df = 6; 1 day vs 6 months ^***^p = 0.0006, df = 6) (Figures 5A,E). We further used TEM to observe the ultrastructural changes of the BBB. We found that vesicle density in the endothelium was significantly increased in the 3–4-week-old and 6-month-old groups (Figures 4F,G). These results indicated that endothelial cell transcytosis was increased during the development of the BBB. In several stroke models, an increase in the number of endothelial cell transcytosis was observed by transmission electron microscopy, along with an increased uptake of the tracer by endothelial cells (Lossinsky and Shivers, 2004). This upregulation of endothelial cell transcytosis increased BBB permeability. Injection of EB was used to assess blood-brain barrier permeability in rats at three time points. We found that the permeability of the blood-brain barrier to EB increased (1 day vs 3–4 weeks *P = 0.0334) with age and the cortex of 6-months-old rats was visually permeable to EB (Supplementary Figure 3).

### Changes in Microvascular Endothelial Tight Junctions During Development

**FIGURE 6:**
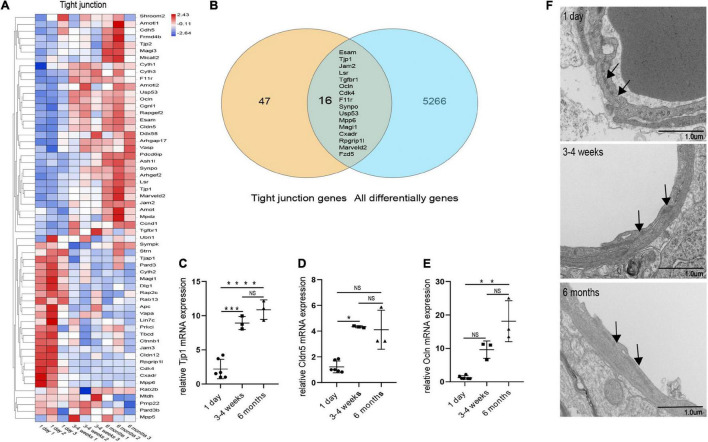
Changes in microvascular endothelial TJs during development. **(A)** Heat map of genes related to BBB TJ components at three different time points. **(B)** Venn diagram of the number of all differentially expressed genes and BBB TJ component genes. All differentially genes represent the differential genes that are compared in pairs between the three groups. **(C)** The expression of tjp1 was examined by qRT-PCR (*n* = 3–6). **(D)** The expression of cldn5 was examined by qRT-PCR (*n* = 3–6). **(E)** The expression of ocln was examined by qRT-PCR (*n* = 3–6). **(F)** Representative images of the ultrastructure of the microvasculature in the 1-day-old, 3–4-week-old and 6-month-old groups. Mean values ± SD, NS, not significant; *p < 0.05, **p < 0.01, ***p < 0.001 and ****p < 0.0001 compared to the Sham group; one-way ANOVA followed by Dunnett’s *post hoc* test.

We performed transcriptome analysis on 47 TJs at three time points. The heat map in Figure 6A shows the changes in TJs expression at these three time points. We further used the Venn diagram to select 16 differentially expressed TJs genes (Figure 6B). We found that tjp1, esam, jam2, and ocln were gradually upregulated during the development of the BBB, while cdk4 and magi1 were gradually downregulated during the development of the BBB in Supplementary Figure 2. The tjp1 gene encodes zo-1, which plays a key role in the development of the BBB. Knockout of the tjp1 gene as early as E8.5 resulted in developmental defects, extensive neuroectodermal tissue apoptosis, vascular development failure, and embryonic death. We used qRTPCR to verify the expression of tjp1, ocln and cldn5 in the cortex microvessels for further verification (Figures 6C–E). We next separated the cerebral cortex microvessels of rats at three time points and extracted the proteins for Western blot analysis. The results showed that, with the development of the BBB, the expression of TJs such as occludin (Figure 5C, 1 day vs 3–4 weeks ^***^p = 0.0009, 1 day vs 6 months ^***^p = 0.0002), claudin5 (Figure 5D, 1 day vs 3–4 weeks ^***^p = 0.0004) and zo-1 (Figure 5F, 1 day vs 3–4 weeks ^**^p = 0.0013; 1 day vs 6 months ^***^p = 0.0003) gradually increased and stabilized (Figure 5B). We further observed under the electron microscope that TJs are short and simple in neonatal brain microvascular endothelial cells. In adult brain microvascular endothelial cells, the particle density rises, and a significant increase in the correlation between TJs and the protoplasmic surface (P-Face) can be observed, which represents BBB continuing to mature (Figure 6F). Cav1–deficient (cav-1−/−) mice had significantly higher degradation of TJ proteins such as occludin, claudin 5, zo-1, and proteolytic activity of matrix metalloproteinase as compared to Cav-1 + / + mice. Conversely, the re-expression of cav-1 in Cav-1−/− mice restored TJs protein expression and reduced matrix metalloproteinase proteolytic activity (Choi et al., 2016). Interestingly, the correlation heat map analysis of transcytosis and TJ gene expression showed that cav-1, cldn5 and tjp1 genes have a strong correlation (Figure 5G). We speculate that under physiological conditions, cav-1 may interact with TJs to further promote the formation and stability of the BBB.

## Conclusion and Discussion

### Identification of the Blood-Brain Barrier Transcriptome

The BBB is located at the level of microvascular endothelial cells in the CNS, which includes capillaries, pre-capillary arterioles and post-capillary venules (Northrop and Yamamoto, 2015; Francisco et al., 2020). Previous gene profiling studies of brain vessels had several limitations, including the use of whole vessel fractions that contain many different cell types, the lack of description of the cerebral endothelial isolation procedures, and separation of the entire brain instead of specific areas (Enerson and Drewes, 2006; Richard et al., 2010; Munji et al., 2019). The vasculature is heterogeneous throughout the central nervous system, and the expression of endothelial junction proteins (occludin, claudin-5) is higher in the white matter (WM) compared to the gray matter (GM) (Gross et al., 1986; Wilhelm et al., 2016). In this paper, we combined enzymatic digestion purification with 40 μm filter to generate a transcriptional profile of highly purified microvascular endothelial cells from the cerebral cortex, and provide a new resource for understanding gene expression in the developing and adult BBB. We used pasteurized pipette mechanical homogenization combined with short-time enzymatic digestion, which can ensure tissue separation without affecting cell viability. These transcripts encode TJ molecules, transporters, signaling cascades, and proteins of unknown function, which together form the highly specialized interface during the development of BBB. The BBB transcriptome database is especially useful for understanding the molecular control of central nervous angiogenesis and the BBB permeability in the developing brain.

### Developmentally Regulated Brain Endothelial Signaling Pathways

Previous studies have confirmed that the brain environment sends signals to brain endothelial cells to form the BBB, but the identity of these signals is largely unknown (Richard et al., 2010). Enriched KEGG pathway analysis in the neonatal groups captured some of the key events, such as “nicotine addiction,” and “axon guidance.” Consistent with our results, axon guidance was found as a key player during the developmental period of the human neocortex (Liu et al., 2016). Enriched KEGG pathway analysis in the 3–4-week-old group captured some of the key events, such as “cytokine-cytokine receptor interaction” or “mapk signaling pathway.” Cytokines play important roles in the physiological response to inflammation and neuroregeneration (Banks and Kastin, 1988; Ba Nks and Kastin, 1996; Pan and Kastin, 2001), and may be also important in the further maturation and maintenance of the BBB. Wnt/beta-catenin signaling is a key regulator of cerebral angiogenesis and BBB formation and maintenance; wnt ligands activate Frizzled receptors and Lrp5/6 complexes on the endothelium in the developing brain (Liebner et al., 2008; Zhou et al., 2014; Sweeney et al., 2019). In this study, the wnt and frizzled genes were involved in the cancer pathway, and these genes may further play a role by activating this pathway.

### Identification of Transcytosis and Tight Junctions During Development

The BBB function continues to mature after birth, but the exact time window is still elusive and may depend on the species (Saunders et al., 2013; Halliday et al., 2016). In the mature stage, the mammalian BBB is stabilized by a highly specialized perivascular structure (Zhen et al., 2015), while the fundamental mechanisms underlying the formation and maintenance of the BBB remain largely unknown. Early research identified two structural features of the microvascular endothelium that underlie the restrictive properties of the BBB: special TJs and low rate of transcytosis (Farquhar and Palade, 1963; Reese and Karnovsky, 1967; Haseloff et al., 2015). Transcytosis in microvascular endothelial cells can be divided into two categories: receptor-mediated transcytosis (RMT), in which the ligand binds to the receptor to mediate endocytosis, and non-selective adsorbent transcytosis (Villaseñor et al., 2017). We found that the RMT required to maintain the BBB were different between the neonatal period and the adult period. Slc7a5, which transports branched chain amino acids through the barrier, was highly expressed in the neonatal period, while slc2a1, which transports glucose, was highly expressed in adulthood (Vivo et al., 1991; Tãrlungeanu et al., 2016). This may indicate that the substances needed to maintain BBB were different between development stages. Cav-1 regulates BBB permeability, and studies have shown that the increased number of endothelial caveolae and the rate of transcytosis were the reasons for the hyperpermeability of the BBB in the early stage of MCAO (Knowland et al., 2014). *Phoneutria nigriventer* spider venom-induced BBB opening is associated with a high expression of caveolae frame-forming cav-1 and the process of internalization and enhanced transcytosis (Soares et al., 2014). Therefore, it is considered that cav-1 can increase the BBB permeability *via* caveolae-based transcytosis. In this study, we found that, as the BBB matures, the expression of cav-1 and the caveolae-based transcytosis increased. Besides, we observed increased permeability to EB in rat cerebral cortex at 3–4 weeks and 6 months. Surprisingly, macroscopically increased EB permeability was seen in rat cortex at 6 months. A recent study found that cav-1-mediated vesicle transcytosis was significantly increased in 19-months-old mice compared with 3-months-old mice (Yang et al., 2020). This study suggests that an increase in permeability may occur in 6-month-old rats or earlier. Next, we assessed changes in tight junctions during blood-brain barrier development. We found the density of TJ particles increases, the inter-endothelial junctions become more extensive, interconnected and complexed, suggesting continued maturation of the BBB. While conventional wisdom has emphasized the importance of tight junctions, recent advances have highlighted the regulation of transcytosis as a crucial factor in limiting BBB permeability under physiological conditions and after pathological assaults (Daneman et al., 2010). Consistent with our findings, in the process of rat cortex development, tight junctions are continuously improved, but endothelial transcytosis increases, and the permeability of the blood-brain barrier increases. Our results suggest that under physiological conditions, low vesicle transcytosis is playing an important role in maintaining the integrity of the blood-brain barrier.

In summary, we have generated a comprehensive dataset describing the transcriptome of the developing BBB, which will provide a valuable resource for understanding the development and function of this crucial barrier, as well as its role of modulating CNS function.

## Data Availability Statement

Raw data of RNA sequencing are available through NCBI Sequence Read Archive database (PRJNA790676), at the following link: https://www.ncbi.nlm.nih.gov/sra/ ?term=PRJNA790676. All other data generated or used during the study appear in the submitted article.

## Ethics Statement

This study on animals was reviewed and approved by Zhengzhou University’s Ethics committee. All animals were treated in accordance with the International Guidelines for animal research. All experimental procedures on animals were approved by and conducted in accordance with Zhengzhou University’s regulations and legal requirements.

## Author Contributions

JWZ contributed to the conception and design. WL and JLZ performed the literature search and drafted the manuscript. JS, CG, RS, and RL were involved in analysis and interpretation of data. HC and YW were involved in review and revision of the manuscript. All authors were blinded to the experimental protocol while performing the experiments and the statistical calculations.

## Conflict of Interest

The authors declare that the research was conducted in the absence of any commercial or financial relationships that could be construed as a potential conflict of interest.

## Publisher’s Note

All claims expressed in this article are solely those of the authors and do not necessarily represent those of their affiliated organizations, or those of the publisher, the editors and the reviewers. Any product that may be evaluated in this article, or claim that may be made by its manufacturer, is not guaranteed or endorsed by the publisher.

## Supplementary Material

Supplementary Figure 1 Overview of cortical microvascular purification protocol. **(A)** Separation of the cerebral cortex. After rat euthanasia, the thin slices of subdural meninges (SDM) on the dorsal surface of the brain were carefully removed using the dissecting microscope to validate the removal. The brain was horizontally cut in half, divided cautiously into cortex and internal structures. Then, it was dissected in cold phosphate-buffered saline (PBS). **(B)** Tissue shredding and digestion. The separated cerebral cortex was cut into 1 mm^3^ tissue pieces and digested for 40 min. **(C)** Separation and purification of cerebral microvessels. Microvessel pellets were resuspended in a 20% BSA-V solution and centrifuged (4°C) for 8 min at 1,000 × *g* in order to remove the top layer, containing myelin and brain parenchymal cells. They were filtered with a 40 μm cell strainer and centrifuged at 1,000 × *g* for 2 min to obtain pure pink microvessel fragments. After mixing them with 1 mL trizol, they were stored at −80°C with liquid nitrogen.

Supplementary Figure 2 The 16 changed TJs genes in Venn diagram (*n* = 3). Mean values ± SD, NS, not significant; **p* < 0.05, ***p* < 0.01, ****p* < 0.001 and *****p* < 0.0001 compared to the Sham group; one-way ANOVA followed by Dunnett’s *post hoc* test.

Supplementary Figure 3 **(A)** Rats were given an intraperitoneal injection of Evan’s blue dye, and their brain cortices were dissected the following day after PBS perfusion. **(B)** Quantification of EB at different timepoints. *n* = 10 per group; Mean values ± SD, NS, not significant; **p* < 0.05, ***p* < 0.01, ****p* < 0.001 and *****p* < 0.0001 compared to the 1-day group; one-way ANOVA followed by Dunnett’s *post hoc* test.
