# Supplementary figures and images for: Both the Complexity of Tight Junctions and Endothelial Transcytosis Are Increased During BBB Postnatal Development in Rats
Source: Front Neurosci. 2022 Apr 28;16:850857. doi: 10.3389/fnins.2022.850857 (PMC9095945; doi:10.3389/fnins.2022.850857)

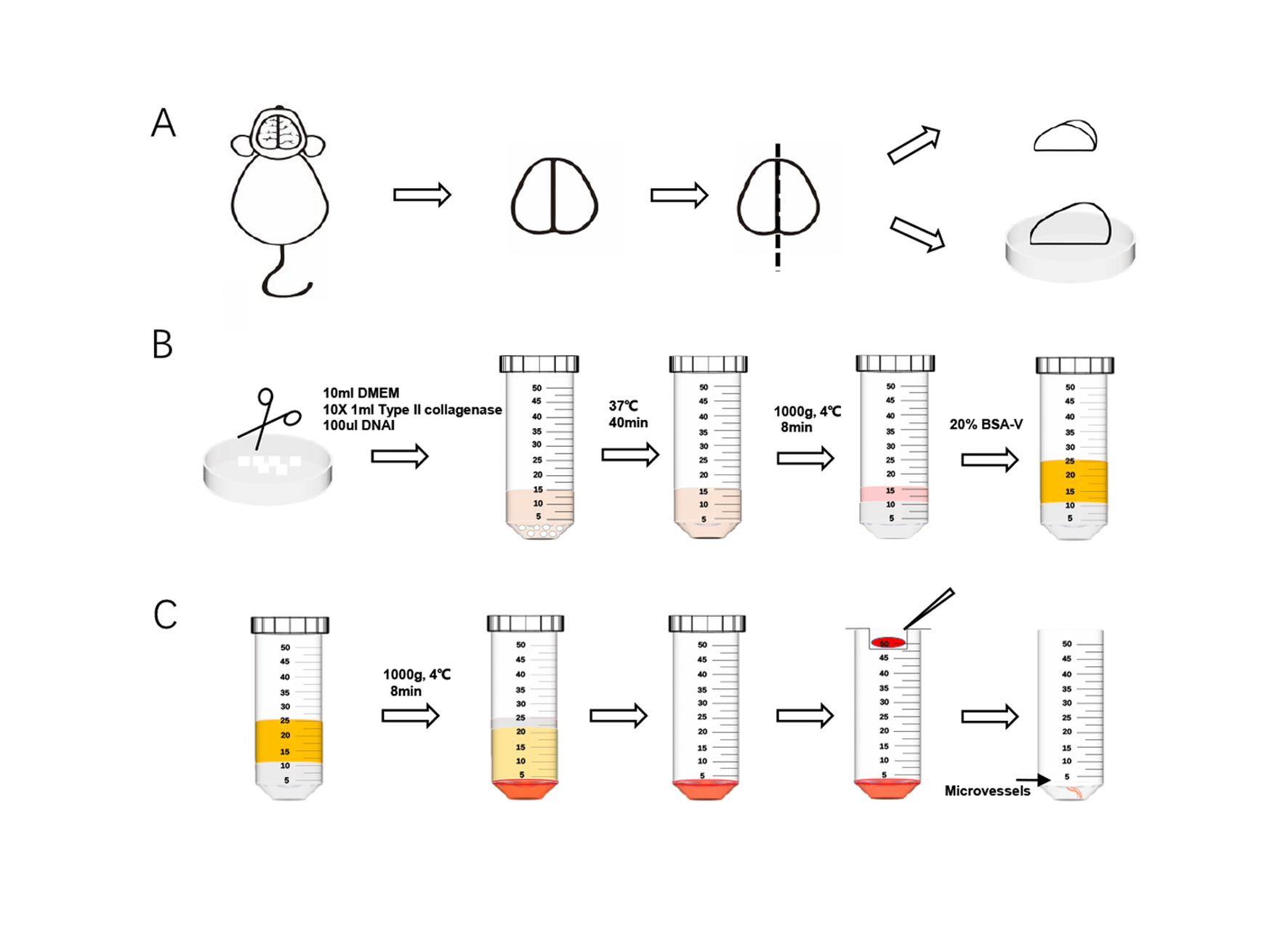

Supplement: Supplementary Figure 1 — Overview of cortical microvascular purification protocol. (A) Separation of the cerebral cortex. After rat euthanasia, the thin slices of subdural meninges (SDM) on the dorsal surface of the brain were carefully removed using the dissecting microscope to validate the removal. The brain was horizontally cut in half, divided cautiously into cortex and internal structures. Then, it was dissected in cold phosphate-buffered saline (PBS). (B) Tissue shredding and digestion. The separated cerebral cortex was cut into 1 mm3 tissue pieces and digested for 40 min. (C) Separation and purification of cerebral microvessels. Microvessel pellets were resuspended in a 20% BSA-V solution and centrifuged (4°C) for 8 min at 1,000 × g in order to remove the top layer, containing myelin and brain parenchymal cells. They were filtered with a 40 μm cell strainer and centrifuged at 1,000 × g for 2 min to obtain pure pink microvessel fragments. After mixing them with 1 mL trizol, they were stored at −80°C with liquid nitrogen. [file Image_1.tif]

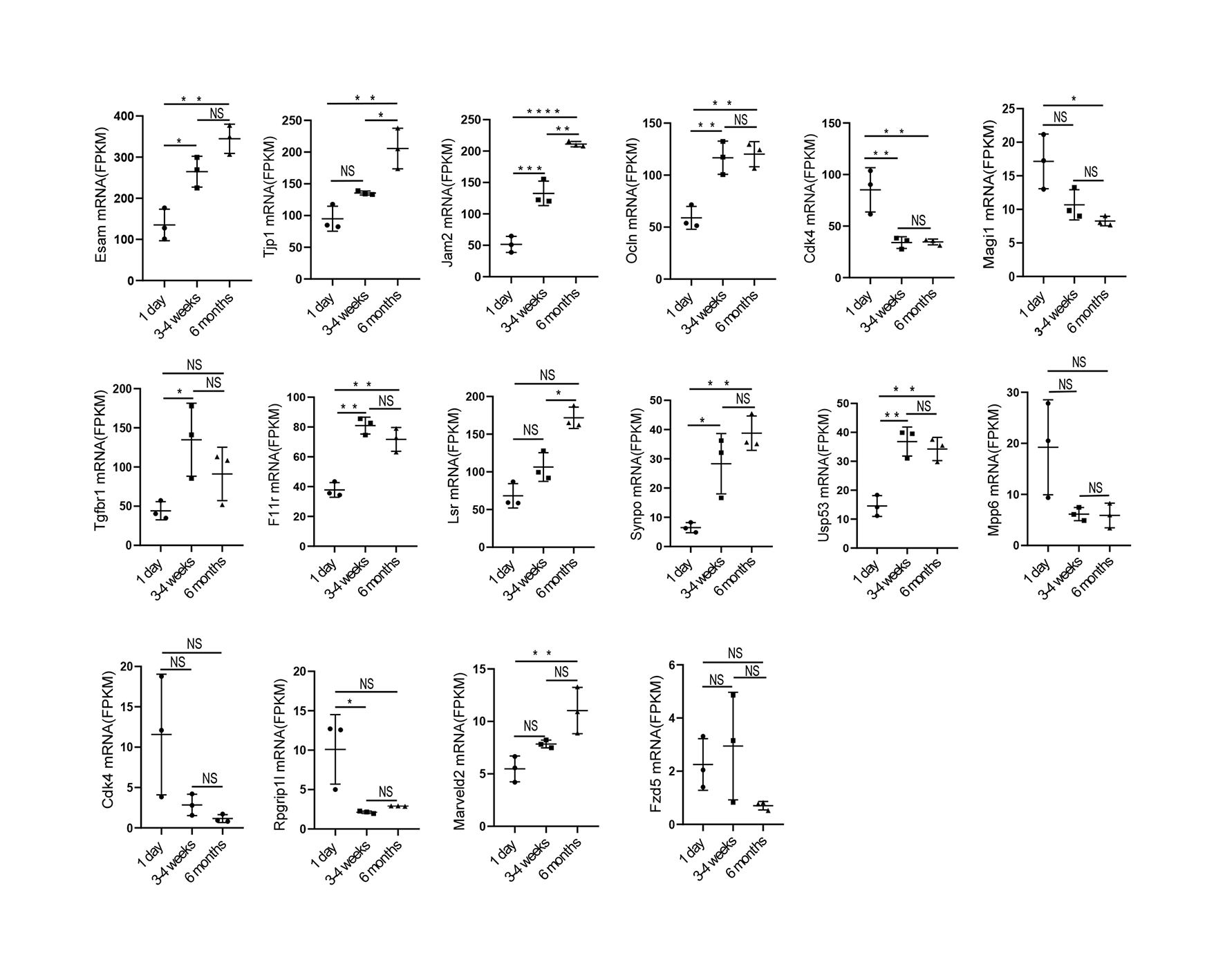

Supplement: Supplementary Figure 2 — The 16 changed TJs genes in Venn diagram (n = 3). Mean values ± SD, NS, not significant; *p < 0.05, **p < 0.01, ***p < 0.001 and ****p < 0.0001 compared to the Sham group; one-way ANOVA followed by Dunnett’s post hoc test. [file Image_2.tif]

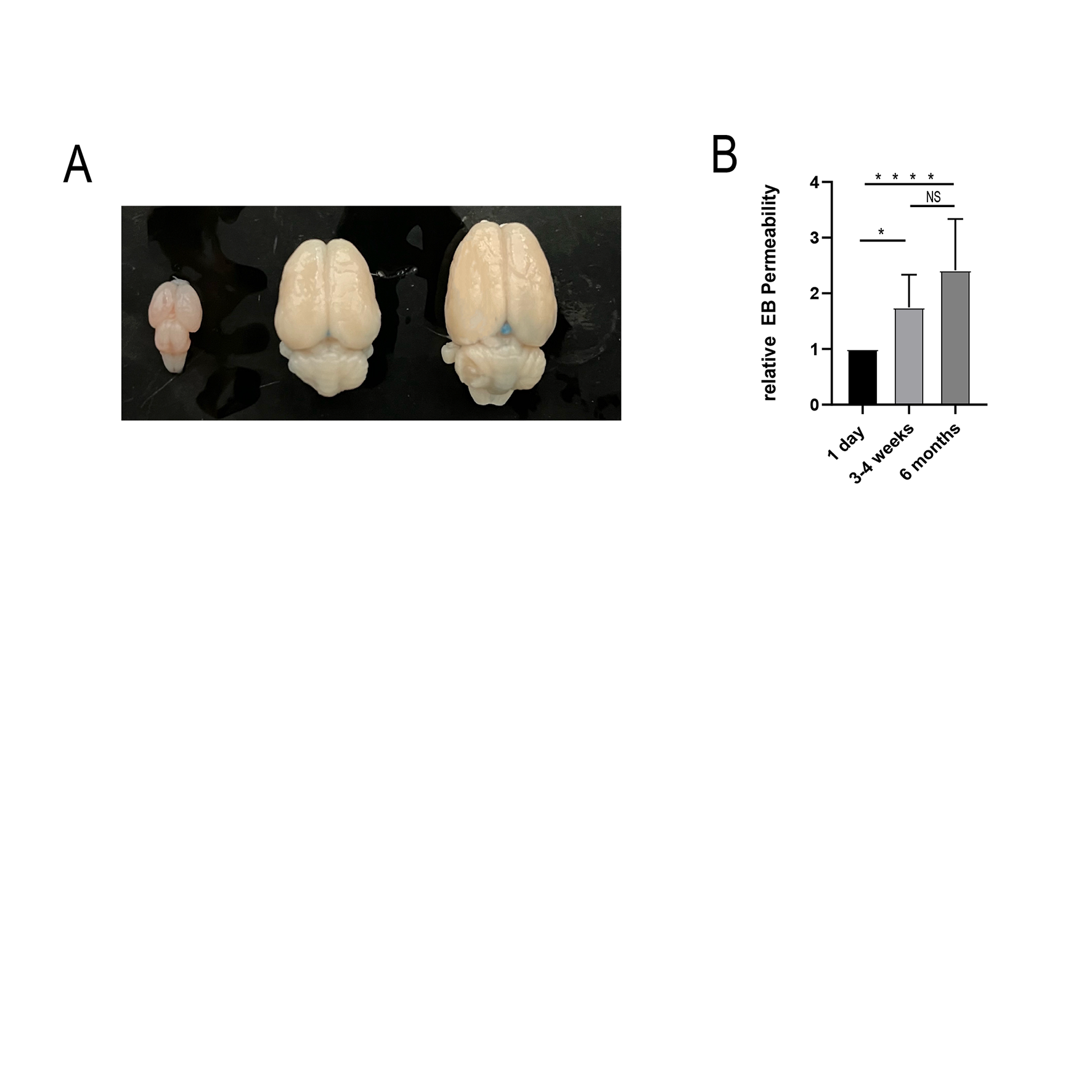

Supplement: Supplementary Figure 3 — (A) Rats were given an intraperitoneal injection of Evan’s blue dye, and their brain cortices were dissected the following day after PBS perfusion. (B) Quantification of EB at different timepoints. n = 10 per group; Mean values ± SD, NS, not significant; *p < 0.05, **p < 0.01, ***p < 0.001 and ****p < 0.0001 compared to the 1-day group; one-way ANOVA followed by Dunnett’s post hoc test. [file Image_3.tif]
